# Multiple SNP Markers Reveal Fine-Scale Population and Deep Phylogeographic Structure in European Anchovy (*Engraulis encrasicolus* L.)

**DOI:** 10.1371/journal.pone.0042201

**Published:** 2012-07-30

**Authors:** Iratxe Zarraonaindia, Mikel Iriondo, Aitor Albaina, Miguel Angel Pardo, Carmen Manzano, W. Stewart Grant, Xabier Irigoien, Andone Estonba

**Affiliations:** 1 Laboratory of Genetics, Department of Genetics, Physical Anthropology and Animal Physiology, University of the Basque Country (UPV/EHU), Sarriena auzoa z/g, Leioa (Bizkaia), Spain; 2 AZTI-Tecnalia, Food Research Unit, Parque Tecnológico de Bizkaia, Astondo Bidea, Edif. 609, Derio (Bizkaia), Spain; 3 Commercial Fisheries Division, Alaska Department of Fish and Game, Anchorage, Alaska, United States of America; 4 AZTI-Tecnalia, Marine Research Division, Herrera Kaia Portualde z/g, Pasaia (Gipuzkoa), Spain; Kunming Institute of Zoology, Chinese Academy of Sciences, China

## Abstract

Geographic surveys of allozymes, microsatellites, nuclear DNA (nDNA) and mitochondrial DNA (mtDNA) have detected several genetic subdivisions among European anchovy populations. However, these studies have been limited in their power to detect some aspects of population structure by the use of a single or a few molecular markers, or by limited geographic sampling. We use a multi-marker approach, 47 nDNA and 15 mtDNA single nucleotide polymorphisms (SNPs), to analyze 626 European anchovies from the whole range of the species to resolve shallow and deep levels of population structure. Nuclear SNPs define 10 genetic entities within two larger genetically distinctive groups associated with oceanic variables and different life-history traits. MtDNA SNPs define two deep phylogroups that reflect ancient dispersals and colonizations. These markers define two ecological groups. One major group of Iberian-Atlantic populations is associated with upwelling areas on narrow continental shelves and includes populations spawning and overwintering in coastal areas. A second major group includes northern populations in the North East (NE) Atlantic (including the Bay of Biscay) and the Mediterranean and is associated with wide continental shelves with local larval retention currents. This group tends to spawn and overwinter in oceanic areas. These two groups encompass ten populations that differ from previously defined management stocks in the Alboran Sea, Iberian-Atlantic and Bay of Biscay regions. In addition, a new North Sea-English Channel stock is defined. SNPs indicate that some populations in the Bay of Biscay are genetically closer to North Western (NW) Mediterranean populations than to other populations in the NE Atlantic, likely due to colonizations of the Bay of Biscay and NW Mediterranean by migrants from a common ancestral population. Northern NE Atlantic populations were subsequently established by migrants from the Bay of Biscay. Populations along the Iberian-Atlantic coast appear to have been founded by secondary waves of migrants from a southern refuge.

## Introduction

European anchovies, *Engraulis encrasicolus*, are widely distributed in near-shore pelagic waters in the Eastern Atlantic from the North Sea and into the Mediterranean and Black Sea and as far south as southern Africa. Populations in the North East (NE) Atlantic and Mediterranean are partitioned into several spawning groups that are isolated from one another by complex shorelines and oceanic regimes [1], [2]. Several biochemical and molecular studies have shown that many of these partitions are congruent with genetic differences between populations [3]–[13]. In some areas, small-scale structure appears among anchovy populations in the Bay of Biscay [12], [13], the North Western (NW) Mediterranean [14]–[16], Adriatic Sea [6] and Black Sea [17]. It is uncertain whether these fine-scale differences are due to adaptive divergence [5], [17], or to cryptic species diversity [14]–[16]. The analysis of mitochondrial (mt) DNA has further resolved two deep matriarchal lineages in European anchovies that appear to reflect ancient isolations, dispersals and colonizations [9], [10], [18]. These lineages, or mtDNA phylogroups, vary greatly in frequency from one location to another, but are not strictly associated with the geographical population groups delimited by nuclear molecular markers [5]–[13]. The historical events that influenced the geographical distributions of these phylogroups have been a topic of debate [9], [19]. Phylogroup A occurs at a high frequency in the Black and Aegean seas and in the eastern Atlantic, but only at intermediate frequencies at most locations in the Mediterranean and NE Atlantic [10]. In contrast, phylogroup B occurs at moderate frequencies at many locations [10]. A mtDNA haplotype frequency shift occurs between Mediterranean and Atlantic populations [9], [10], and this shift is echoed by a shift in microsatellite allele frequencies [13]. The mechanisms leading to high abundances of phylogroup A in disjunct locations are still unresolved. So far, no morphological or adaptive differences have been detected between individuals in phylogroups A and B [11].

While migration, genetic drift and adaptive selection may explain much of the genetic structure of anchovy populations, deeper genetic partitions with coalescences dating to tens of thousands of years more likely reflect ancient climate events during the Pleistocene ice-ages. During each ice age, continental glaciers expanded across North America and Eurasia, leading to lower ocean temperatures and sea levels [20]. These drops in sea level gave rise to expanded shorelines, and in some places to the appearance of land barriers that severed connections between marine populations [21]. As temperatures dropped, populations likely tracked optimal habitats as they shifted toward lower latitudes [22]. Populations moved poleward and dispersed into suitable coastal habitats as mid-latitude waters warmed and coastal barriers receded with rising sea levels. These ocean-climate shifts greatly influenced anchovy populations in the NE Atlantic and Mediterranean and potentially imprinted the genetic structures of contemporary populations. Hence, the examination of genetic variability can potentially provide insights into the population histories of European anchovies.

Previous studies of European anchovies have been limited in two ways. First, most studies of European anchovies have been regional, where samples have been restricted to one of the many sub-basins in the Mediterranean Sea. Populations in the North Sea and Bay of Biscay and along the Iberian-Atlantic coast have received less attention. The analysis of these populations will provide a deeper understanding of population structure and history. Second, the power of some molecular markers to detect some aspects of population structure is weak. Most studies have used neutral molecular markers, which are interpreted in terms of gene flow and genetic drift (population size) [23]. These markers have been important in addressing problems in fishery management and conservation. However, some markers, including allozymes and mtDNA associated with coding genes, may sometimes be influenced by natural selection and may not be suitable for estimating population parameters [24], [25]. Moreover, mtDNA may have limited value, because linkage propagates the same population signal among genes. Additionally, a large evolutionary variance among loci limits the ability of a single marker to discern many aspects of population history, because a single locus represents only one of a large number of possible evolutionary realizations [26].

The goal of this study was to examine variation at several SNP loci to better resolve the genetic structure of anchovy populations in the NE Atlantic. A multi-locus approach is more likely to yield a fuller snapshot of population structure than the analysis of a single marker [27], [28]. While microsatellites have been used in the past few decades, the analysis of a large number of SNP loci can provide additional insights into anchovy population structure [29]. A high-resolution view of anchovy genetic population structure will lead to a better understanding of the effects of Pleistocene ocean-climate shifts on anchovy populations and will provide a stronger foundation for fishery management. The chief value of genetic data to management is the identification of demographically independent populations with different patterns of recruitment, mortality and productivity [30].

## Materials and Methods

### a) Samples and DNA Extraction

Anchovies were provided either by commercial vessels or by oceanographic institutes that collected the samples during scientific acoustic surveys (BIOMAN, PELGAS, ECOCADIZ, ECOMED, PELACUS). All surveys followed local regulations and guidelines for such research. Anchovies were collected following fishing without unnecessary suffering of the animals and following usual procedures. No experimentation with animals was performed. No other ethical issues applied to the present research project.

**Table 1:** *Engraulis encrasicolus* sample description.

Sample number	Location	*N*	Latitude	Longitude	Date
***North Sea and English Ch.***					
1	Kiel	29	54°54′60″N	10°10′0.00″E	Nov-06
2	Denmark	19	57°8′30.00″N	11°34′0.00″E	Jul-07
3	Germany	12	54°26′1.92″N	6°29′39.42″E	Apr-07
4	Scotland-1	13	56°8′23.08″N	2°19′36.35″E	Feb-09
5	Scotland-2	29	58°5′10.20″N	1°08′42.00″W	May-09
6	English Channel*	25	50°11′42.83″N	4°16′32.36″W	2002–2007
***Bay of Biscay***					
7	Bisc-5029	26	45°52′28.77″N	1°52′23.81″W	May-08
8	Bisc-5020	28	44°35′48.00″N	1°55′23.00″W	May-09
9	Bisc-5001	49	43°21′0.00″N	2°12′36.21″W	May-09
***East Atlantic Coast***					
10	Galicia	30	42°31′48.00″N	8°56′24.00″W	Mar-10
11	Aveiro	28	40°42′30.00″N	8°39′36.00″W	2008
12	Portugal-S	33	38°35′45.77″N	9°21′0.00″W	Feb-08
13	Gulf of Cadiz	60	36°32′13.20″N	6°28′24.00″W	Apr-09
14	CanaryIslands*	29	27°42′58.58″N	15°38′42.29″W	May-07
15	South Africa	30	34°0′0.00″S	18°0′0.00″E	Sep-09
***Mediterranean Sea***					
16	Alboran Sea*	68	36°31′55.36″N	4°2′14.12″W	Oct-09
17	Delta	31	40°33′4.51″N	0°53′33.42″E	2007
18	Tarragona	26	40°52′60.00″N	1°10′0.00″E	Mar-09
19	Adriatic Sea*	30	42°49′44.91″N	15°28′19.31″E	Oct-07
20	Aegean Sea	31	40°36′30.00″N	24°9′50.00″E	Jul-08
	Total	626			

Location, sample size (*N*), latitude, longitude and sampling date.

* Approximate coordinates.

**Figure 1:**
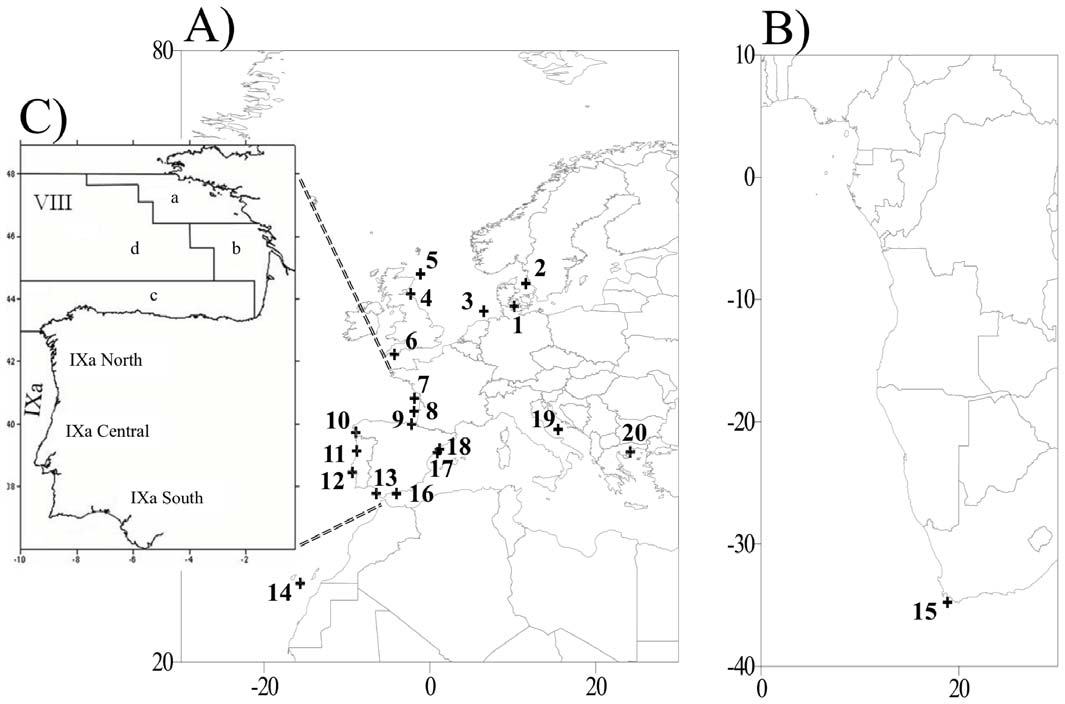
Map showing locations of samples of European anchovies. A) North Sea to Canary Islands samples (1–14) along with Mediterranean samples (16–20). B) South African sample (15). C) Geographical limits of ICES Divisions (VIIIa, VIIIb, VIIIc, VIIId and IXa) (modified from Caneco et al. 2004).

A total of 626 anchovies (mean *N* = 30) were collected from the entire geographic range of this species and its spawning areas (Table 1, Fig. 1). Five locations (*N* = 188) were sampled in the Mediterranean Sea along an east-west axis. Several locations were sampled in the Eastern Atlantic (*N* = 443), including 127 fish from the North Sea and English Channel (6 locations), 106 fish from the Bay of Biscay (3 locations) and 210 fish from Galicia to South Africa (7 locations). Most samples were collected from 2007 to 2009. Genomic DNA was extracted from muscle tissue using the DNeasy 96 Tissue Kit (Qiagen), following manufacturer’s protocol. A specific protocol was used (www.ambion.com/techlib/misc/genomicDNA_rnalater.html) for tissues preserved in RNA*later*® (Applied Biosystems), as was the case for North Sea and English Channel samples.

### b) SNP Selection and Genotyping

The 90 novel SNPs, including 75 nuclear (n) DNA and 15 mtDNA SNPs, described by Zarraonaindia et al. included in Molecular Ecology Resources Primer Development Consortium et al. [31] were considered for this study. Five nuclear markers exhibiting significant departures from Hardy-Weinberg expectation (HWE) [31] were discarded. Genotypic Disequilibrium tests, within and among sequenced fragments, were performed for the remaining 70 SNPs using GENEPOP 4.0 [32] with rejection probabilities of *P*<0.001. Linked SNPs were phased into haplotypes using the Bayesian statistical method implemented in PHASE 2.1 [33]. Haplotypes were reconstructed by location to avoid biases from population structuring. Missing genotypes were classified as null genotypes to avoid haplotype reconstruction errors. Only one SNP or haplotype block was chosen for each sequenced fragment to ensure maximum independence among the markers. Markers showing the largest heterozygosities per fragment were given preference. This yielded 49 SNP markers, including 26 individual SNPs and 10 haplotype blocks (Table S1). We additionally tested for natural selection with BayeScan [34] in samples grouped by geographical area. We calculated Bayes factors (BF) to identify candidate loci by using 20 pilot runs of 5000 iterations and an additional burn-in of 50,000 iterations, for a total of 100,000 iterations (sample size of 5000 and thinning interval of 10). Locus specific BFs are the ratio of the posterior probabilities of selection and neutrality, given the data. The marker representing haplotype block KLN-332-(144-444) (Table S1) presented a log_10_BF value >2 corresponding to decisive evidence for selection (BayeScan v2.0 User Manual). Hence, it was discarded from the final SNP panel of 47 nDNA SNPs, representing 35 independent markers including 26 individual SNPs and 9 haplotype blocks. We used FSTAT 2.9.3 [35] to estimate the number of alleles, expected and observed heterozygosity and *F*_IS_ for each sample.

The 626 anchovies from 20 localities were screened for the 47 nDNA SNPs panel and 15 mtDNA SNPs with *TaqMan*® *OpenArray*™ genotyping system. DNA concentrations and reactions for amplification and detection of the SNPs followed the *TaqMan*® *OpenArray*™ *Genotyping System User Guide.* Genotypes were scored using Autocaller 1.1 (Applied Biosystems).

### c) Statistical Analysis

Nuclear SNPs were examined in three ways. First, population structure was inferred from individual assignments based on the analysis of the nuclear SNPs using the Bayesian model-based clustering algorithm implemented in STRUCTURE 2.3.2 [36]. This program uses Hardy-Weinberg proportions and gametic disequilibrium to cluster individuals into *K* groups. Ten independent runs were conducted for each value of *K* (*K* = 1–10) using 500,000 Markov Chain Monte Carlo iterations, after a burn-in of 50,000 iterations. We assumed a mixed ancestry model and correlated allele frequencies [37] and used a *locprior* model. We used CLUMPP 1.1.2 [38] to determine optimal assignments of individuals to clusters, maximizing similarity between the different STRUCTURE replications. The most likely number of populations (*K*) that best explained the pattern of genetic variability was evaluated using the approach of Evanno et al. [39]. Graphical output of individual membership coefficients in each cluster was created with DISTRUCT 1.1 [40].

Second, we estimated genetic distances between samples and used these distances in additional analyses. Reynolds genetic distance [41] was estimated between samples from allele frequencies over loci using POPULATION 1.2.28 [42]. This matrix was used to construct a Neighbour-Joining (NJ) tree for which topological confidence was evaluated with 1000 bootstrap replicates. Isolation by distance was tested by searching for a correlation between genetic and geographical distance matrices. Geographical distances (km) were calculated as the shortest path between sample locations inside the 1000 m isobaths and by considering the known spatial distribution of the European anchovy. The matrix was obtained using the spatial analysis tool *Path Distance* implemented in ArcGis 9.2. Matrix comparisons by Mantel’s method were carried out with the program ZT [43], and significance was determined with 10,000 iterations.

Lastly, genetic differentiation among samples was estimated with the unbiased fixation index (*F*_ST_) [44] using FSTAT [35], [45]. Confidence intervals for *F*_ST_ were determined by jackknifing, and statistical significance was determined with 15,000 permutations. We used the Bonferroni correction probabilities for multiple tests [46]. Population groups were defined by non-significant values of mean *F*_ST_ between samples and by significant values of *F*_ST_ with other populations [30].

The number of SNP mtDNA haplotypes (*n*_h_), haplotype diversity (*h*), and Tajima’s *D* [47] and Fu’s *F*_S_ [48] measures of neutrality were estimated with DNAsp 5 [49]. The statistical significances of *D* and *F*_S_ were tested with 1000 coalescent simulations. Mutational relationships among haplotypes were represented by a median-joining (MJ) network [50] constructed with NETWORK 4.2 (http://www.fluxus-technology.com). The SNPs *Cyt-b-318*, *Dloop-323* and *Dloop-336* were not used to construct the network, because their high levels of polymorphism would reduce the resolution among haplotypes.

## Results

### a) Nuclear DNA Analysis

**Figure 2:**
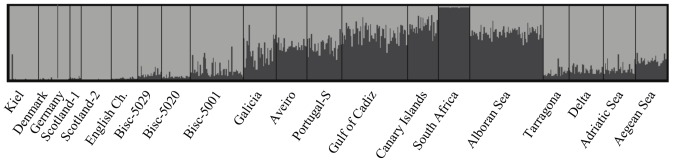
Individual clustering analysis obtained by STRUCTURE analysis of 626 European anchovies for *K* = 2. Each vertical bar represents an individual and the different geographic areas that were sampled are separated by vertical black lines. The color proportions of each bar correspond to the individual’s estimated membership fractions to each of the clusters (cluster membership coefficient).

STRUCTURE analysis of 626 anchovies yielded the most likely partition of *K* = 2. Membership in these two groups varied latitudinally (Fig. 2). One group included samples from the North Sea and English Channel, the Bay of Biscay and the Mediterranean (excluding the Alboran Sea). The other group was represented by anchovies in South Africa and encompassed samples from eastern Atlantic locations extending from Galicia to South Africa, but also including the Alboran Sea.

**Figure 3:**
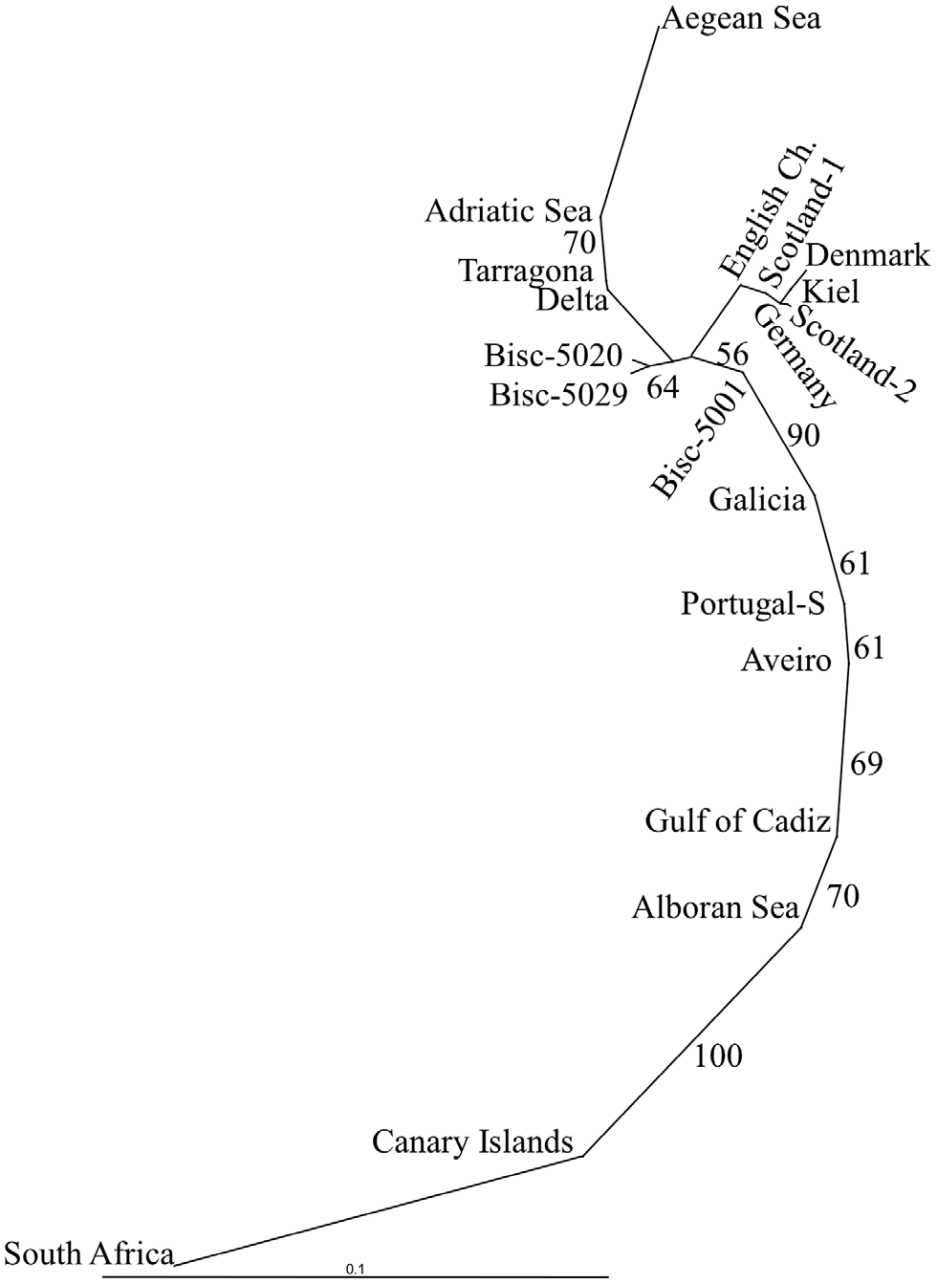
Neighbour-Joining tree of Reynolds distances between samples of European anchovies. Topological confidence obtained by 1000 bootstrap replicates. Only bootstrap values larger than 50% are shown.

A similar pattern of population differentiation appeared in genetic distances between populations. The NJ tree of Reynolds genetic distances also showed two major groups supported by a 90% bootstrap value (Fig. 3). The first group consisted of NE Atlantic samples, including those from the North Sea, Bay of Biscay and the Mediterranean, except the Alboran Sea. The second group consisted of Atlantic samples arranged latitudinally in the tree from Galicia to South Africa, but also including the Alboran Sea. The test for isolation by distance (IBD) among populations was significant (Mantel’s test: *r* = 0.771, *P* = 0.0001; matrix 20×20). IBD among the NE Atlantic samples from the Bay of Biscay to the Canary Islands (including the Alboran Sea) was highly significant (*r* = 0.903, *P* = 0.0007; matrix 7×7). The addition of the distantly located sample from South Africa lowered the correlation between geographical distance and genetic distance (*r* = 0.789, *P* = 0.0001; matrix 8×8).

Pairwise *F*_ST_ ranged from 0.04, between two samples from the Bay of Biscay, to 0.318 between samples from Denmark and South Africa, and averaged 0.078±0.012 (Table 1). No significant *F*_ST_ values appeared between samples collected in different years at nearby locations. For example, *F*_ST_s between samples from Delta (2007) and nearby Tarragona (2009) in the NW Mediterranean and between samples from the North Sea (2006–2009) were not significant. Based on these pairwise *F*_ST_ values, the original 20 samples were pooled into 10 groups, which showed within-group homogeneity but between-group heterogeneity. The largest amount of divergence between these 10 groups was *F*_ST_ = 0.270±0.053 between the South African and Aegean Sea samples (Table 2). Importantly, a significant amount of divergence (*F*_ST_ = 0.013±0.007) was detected between samples from nearby locations in the Bay of Biscay (BISC-1: Bisc-5020, Bisc-5029; and BISC-2: Bisc-5001), which were placed into two different pooled groups. Both pooled groups (BISC-1 and BISC-2) showed a larger amount of divergence from the populations in the North Sea and English Channel pooled group (NSEC), (*F*_ST_ *= *0.031±0.013 and *F*_ST_ *= *0.027±0.008, respectively) than from NW Mediterranean samples (NWMD) (*F*_ST_ *= *0.020±0.011 and *F*_ST_ *= *0.024±0.013, respectively).

**Table 2:** *F*_ST_ values between the 10 homogeneous population groups of European anchovies.

	NSEC	BISC1	BISC2	CIAT	SIAT	CAN	SAF	NWMD	ADR	AEG
**NSEC**		0.013	0.008	0.016	0.023	0.033	0.042	0.014	0.022	0.041
**BISC1**	0.031		0.007	0.014	0.020	0.026	0.035	0.011	0.019	0.036
**BISC2**	0.027	0.013		0.008	0.014	0.023	0.033	0.013	0.019	0.036
**CIAT**	0.063	0.053	0.022		0.005	0.011	0.017	0.020	0.017	0.023
**SIAT**	0.114	0.103	0.069	0.020		0.015	0.020	0.029	0.025	0.030
**CAN**	0.169	0.149	0.110	0.045	0.026		0.011	0.034	0.032	0.031
**SAF**	0.247	0.241	0.198	0.105	0.073	0.042		0.051	0.046	0.053
**NWMD**	0.046	0.020	0.024	0.056	0.098	0.137	0.235		0.008	0.013
**ADR**	0.041	0.042	0.039	0.053	0.085	0.141	0.233	0.014		0.011
**AEG**	0.092	0.088	0.086	0.079	0.106	0.163	0.270	0.038	0.027	

Pairwise *F*_ST_ values between population represented below diagonal and pairwise standard error (SE) above diagonal. All comparisons in the table were significant (*P*<0.05) after Bonferroni correction of rejection probabilities. Population group abbreviations: NSEC (Kiel, Denmark, Germany, Scotland 1 and 2, English Channel); BISC-1 (Bisc-5029 and Bisc-5020); BISC-2 (Bisc-5001); CIAT (Central Iberian-Atlantic: Galicia, Aveiro, Portugal-S); SIAT (Southern Iberian-Atlantic: Gulf of Cadiz, Alboran Sea); CAN (Canary Islands); SAF (South Africa); NWMD (North-western Mediterranean: Tarragona, Delta); ADR (Adriatic Sea); and AEG (Aegean Sea).

### b) Mitochondrial DNA Analysis

**Figure 4:**
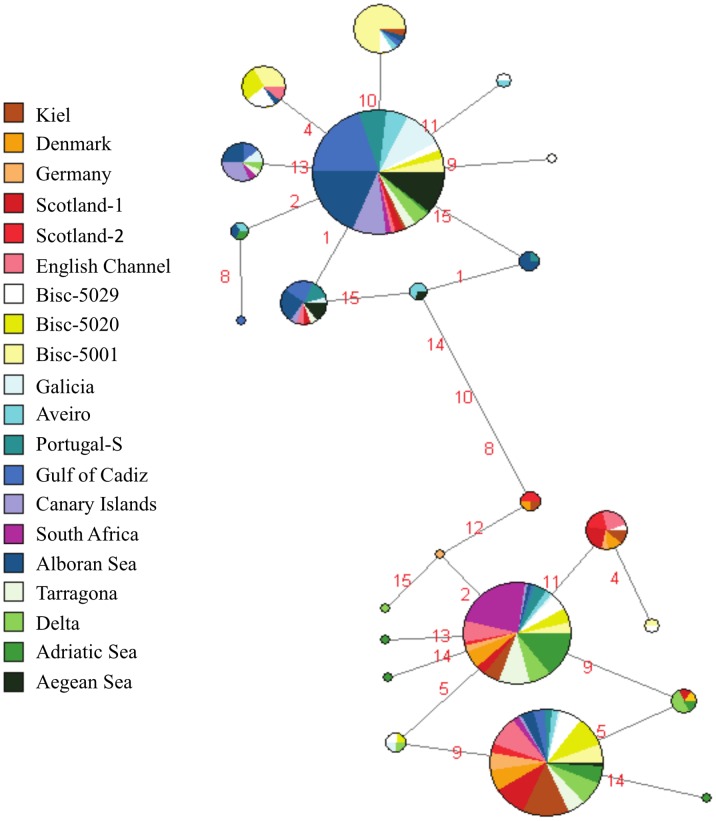
Median Joining Network of haplotypes in European anchovies defined by 12 of the 15 mitochondrial DNA SNPs. (*Cytb-318*, *Dloop-323* and *Dloop-336* were given a weight of 0). Phylogroups A and B were separated by 3 mutational steps, while other haplotypes were separated by 1 mutational step. Numbers along the branches specify the mutated SNP: 1) *CYTb-60*, 2) *CYTb-156*, 3) *CYTb-318*, 4) *CYTb-516*, 5) *CYTb-534*, 6) *Dloop-323*, 7) *Dloop-336*, 8) *Dloop- 486*, 9) *Dloop-568*, 10) *mt-12S-358*, 11) *mt-12S-390*, 12) *mt-12S-454*, 13) *mt-16S-1176*, 14) *mt-16S-1180*, 15) *mt-16S-1227*.

The 15 mtDNA SNPs defined 55 haplotypes, but only 23 haplotypes after excluding the highly polymorphic SNPs, *Cyt-b-318*, *D-loop-323* and *D-loop-336*. The MJ Network showed two phylogroups, A and B, separated by 3 mutational steps, including a transition, a transversion and an indel (Fig. 4). Haplotype diversity in phylogroup A (*h* = 0.825, SD = 0.012) was similar to haplotype diversity in phylogroup B (*h* = 0.829, SD = 0.014). Phylogroup A displayed a star-like haplotype network, and both Tajima’s *D* and Fu’s *F*_S_ were negative, but not significant (*D* = −0.605; *P*>0.10 and *F*_S_ = −19.950; NS). In contrast, the phylogroup B network was more reticulated, with two predominant haplotypes. Neither *D* (*D* = 0.171; *P*>0.10) nor *F*_S_ (*F*_S_ = −15.085; NS) was significant. The mismatch distribution for both phylogroups was unimodal (not shown). In contrast, the two phylogroups together showed a bimodal mismatch distribution, with a positive and significant Tajima *D* value (*D* = 3.155, *P*<0.01). Both phylogroups appeared in each sample, but at different frequencies (Table 3). Phylogroup A was common in the Atlantic, from Galicia to the Canary Islands and in the Alboran and Aegean seas. Phylogroup B predominated in the North Sea and English Channel, Adriatic Sea and southern Africa. Phylogroups A and B occurred at similar frequencies in samples from the NW Mediterranean and the Bay of Biscay. Moderate haplotype diversities appeared in samples from Galicia (*h* = 0.606) and South Africa (*h* = 0.669), whereas the largest diversities appeared in the Bay of Biscay (Bisc-5029: *h* = 0.929; Bisc-5020: *h* = 0.912) and English Channel (*h* = 0.913).

**Table 3:** Mitochondrial DNA summary statistics for samples of European anchovies.

Locality	*N*	*n*_h_	*h*±(SD)	%A	%B
Kiel	28	11	0.825±0.061	11	89
Denmark	15	7	0.876±0.052	7	93
Germany	11	6	0.727±0.144	10	90
Scotland-1	11	7	0.873±0.089	9	91
Scotland-2	24	10	0.841±0.062	21	79
English Ch.	25	11	0.913±0.029	19	81
Bisc-5029	29	16	0.929±0.029	45	55
Bisc-5020	27	11	0.912±0.025	39	61
Bisc-5001	45	16	0.875±0.036	69	31
Galicia	29	8	0.606±0.100	90	10
Aveiro	23	12	0.905±0.041	78	22
Portugal-S	28	10	0.849±0.046	79	21
Gulf of Cadiz	60	14	0.812±0.031	92	8
Canary Island	28	8	0.854±0.032	93	7
South Africa	30	9	0.669±0.091	13	87
Alboran Sea	68	19	0.851±0.027	93	7
Tarragona	26	10	0.874±0.034	35	65
Delta	33	15	0.892±0.034	33	67
Adriatic Sea	27	13	0.863±0.051	11	89
Aegean Sea	32	7	0.758±0.048	97	3

Sample size (*N*), number of haplotypes (*n*_h_), haplotypic diversity with standard deviation (*h*±SD*)* and phylogroup frequencies (%A and %B).

## Discussion

The present study combines the use of mitochondrial and nuclear SNP markers, together with a large coverage of the distributional range of the European anchovy to provide a deeper understanding of the factors shaping the genetic population structure of the species and to disentangle historical from ecological factors reflected in contemporary populations. On the one hand, the analysis of maternally inherited, non-recombining mtDNA complements nuclear SNPs and provides insights into ancient events that have produced deep genetic/genomic partitions in European anchovies. On the other hand, nuclear SNPs enhance the power to detect population divergences resulting from genetic drift.

When comparing the resolving power of nDNA SNPs with other types of markers, such as microsatellites, the use of 4–12 nuclear SNPs is expected to have the same resolving power as a microsatellite locus [29]. In addition, the use of blocks of linked SNPs, each treated as a haplotype, yields genotypes with properties similar to microsatellite genotypes [51]–[53]. In the present study, 47 nuclear SNP representing 35 independent makers have been used and among them 9 markers consisted of haplotype blocks. These SNP markers provided *F*_ST_ values that were 7–8 times larger between samples than *F*_ST_ values estimated with 7 microsatellite loci [13]; the average microsatellite *F*_ST_ between the Bay of Biscay, Gulf of Cadiz and Mediterranean anchovy samples was 0.029±0.013 [13], but for nuclear SNPs the average *F*_ST_ was 0.224±0.057 (present study). Furthermore, microsatellites were not powerful enough to find genetic differences among one Bay of Biscay sample and western Mediterranean samples [13], whereas SNP markers provided enough power to detect significant differences among the samples of both regions. Hence, our study achieved greater resolution of population structure with SNP markers than would have been possible with other markers.

Regarding the sample design, the strategy followed was successful to reveal both, small and large spatial scale population structure. First, samples were taken from oceanic and near-shore populations to test for possible genetic differences between anchovies showing different life-history patterns. Second, we concentrated our sampling effort in the NE Atlantic, an area that had previously not been well studied. Populations of anchovies in northern areas of the NE Atlantic were of particular interest, because these areas have been open to colonization in only the past few thousand years [21]. Third, samples collected in the Mediterranean and farther afield in southern Africa provide the opportunity to compare our results with those of other studies and to construct an overall picture of anchovy population structure. Finally, we also considered the temporal dimension. Even if we did not sample the same population in different years, the lack of significant frequency differences between nearby populations over a 2–3 year period gives us confidence in our spatial analyses.

### Population Structure and Management Implication

On large geographical scales, two genetic groups of populations were identified (Fig. 2, 3) that appear to be associated with different oceanic regimes. One group includes populations in the North Sea, Bay of Biscay and Mediterranean Sea that inhabit wide-shelf areas characterized by larval-retention mechanisms. These anchovies spawn and overwinter in more oceanic offshore areas [54]–[59]. The ecology of anchovies in this group reflects the classic ‘ocean triads’ model of a productive environment that is also conducive to egg and larval retention [1]. The other group included populations extending from Galicia on the Iberian Peninsula to at least the Canary Islands, but also included southern Africa. Populations in these areas inhabit narrow-shelf waters associated with upwelling. In contrast to the first group, these anchovies spawn and overwinter in coastal areas to avoid offshore advection driven by upwelling [60]–[64]. Populations from the later group are characterized by isolation by distance (IBD), common among populations of small- to medium-sized pelagic fishes with life histories similar to those of European anchovies [65], [66]. The prevalence of IBD in European anchovies and in these marine species implies an approach to equilibrium between migration and genetic drift [67].

On geographical scales of hundreds of kilometers, genetic data resolved at least ten homogeneous population groups (Table 2) that likely arose after the last glacial maximum as a consequence of limited gene flow between groups, or may reflect secondary contact between previously isolated populations. These genetic groups differ in some cases from the stocks defined for management in the species.

Genetic homogeneity among anchovy samples in the North Sea-English Channel area and genetic divergence between this northern-most population group (NSEC, Table 2) and those in the Bay of Biscay anchovies indicate that North Sea-English Channel anchovy populations should be managed as an independent stock unit. Anchovies have not been historically abundant in the North Sea region, but abundances have increased in the last few years, possibly in response to oceanic warming [58], [68] and may support fisheries in the future.

Regarding southern European populations, the genetic division found in the east Iberian Atlantic area appears to correspond to morphological differences. Caneco et al. [69] reported morphometric differences between anchovies from the Gulf of Cadiz and the Portugal area, suggesting that these differences may be due to adaptation to environmental differences between areas. Asynchronous abundances also indicate demographic independence between populations in these two areas. Large populations in Galicia and Portugal historically supported large harvests until the early 1960s when these populations declined [70], [71]. A southern center of abundance is located in the Gulf of Cadiz, which presently supports a large fishery. However, currently these two groups (Portugal and Gulf of Cadiz anchovies) are managed as a single stock (ICES Division IXa; Fig. 1) [72].

In the Mediterranean, our sampling does not allow us to define the geographical boundaries between the four genetically distinct groups (Table 2), but our findings agree with previous genetic studies [5]–[13]. These populations roughly correspond to the four major centers of anchovy abundance and areas conducive to spawning, larval retention and growth, the ‘ocean triad hypothesis’ proposed by Agostini & Bakun [1] based on a consideration of oceanic processes. A close relationship between anchovies in Alboran Sea and Atlantic anchovies has been reported (Table 2, Fig. 2, 3), suggesting that the Almeria-Oran front is a barrier to dispersal for anchovies [10]–[12]. The latter genetic similarity throws a new light on the harvest management of anchovies in the Alboran area. Under the current management policy, Alboran Sea anchovies are grouped with NW Mediterranean anchovies. However, the results of our study of SNPs and a previous study of allozymes [12] show that the anchovies in the Alboran Sea are more closely related to populations in the adjacent Gulf of Cadiz. These two stocks together, therefore, represent a more meaningful management unit and should be treated as a single stock.

Small, but significant, amounts of divergence appeared between anchovy populations in the Bay of Biscay (Table 2) that are presently managed as a single stock [72], [73] (ICES division VIII, Fig. 1). Genetic heterogeneity among populations in the Bay of Biscay previously appeared in studies of allozyme [12] and microsatellite [13] variability. The extent of differentiation among populations in the Bay of Biscay is not well understood, nor are the mechanisms producing and maintaining this heterogeneity. One possibility is that these populations reflect divergence in life-history patterns [74], or are an undescribed taxon [15]. However, the present genetic result implies demographic independence between these populations that should be incorporated into harvest management strategies.

### Historical Biogeography

Genetic relationships among populations within regions are here interpreted to reflect ancestral relationships that have been overlain with patterns of contemporary gene flow, but not to the extent that historical signals of dispersal have been erased. The NE Atlantic experienced strong ocean-climate shifts during the Pleistocene. Periodic drops in sea surface temperatures and lower sea levels led to the elimination of anchovy habitats in northern areas during the last glacial maximum about 18,000 years ago. Even after continental glaciers largely receded, a land bridge existed between the British Isles and continental Europe until about 7500 years ago [21] that blocked dispersals of anchovies into northern areas in the NE Atlantic. Previous genetic results for mtDNA [9] show that some populations in the Bay of Biscay are more closely related to populations in the NW Mediterranean. This geographically unusual relationship is confirmed here with nuclear SNP markers. The relationship cannot be explained by contemporary gene flow, because genetically differentiated populations inhabit the intervening areas along a potential dispersal route. This similarity more likely reflects ancient founding events by colonists from a common ancestral population. The hypothesis of a common shared ancestor had been previously suggested [9], [10], [13], [19], but the inclusion of a large number of samples in the NE Atlantic and the analysis of a large number of markers in our study allows us to refine this hypothesis.

Other regional relationships also appear to reflect ancient dispersals and founder events. For example, small values of *F*_ST_ between northern North Sea-English Channel and some populations in the Bay of Biscay indicate a close genetic relationship between these groups that may reflect historical biogeographic relationships. Present-day northern populations and those in the Bay of Biscay have increased in abundance since the 1990s [58]. Beare et al. [58] proposed that the recent expansion of anchovies in the North Sea was due to a northern shift in the distributions of southern populations because of ocean-climate warming. However, Petitgas et al. [68] countered that population growth in the North Sea was an expansion of remnant populations in response to a widening of their thermal habitat and to higher levels of ocean productivity. The small, but significant, differences between northern populations and those in the Bay of Biscay support the latter hypothesis and suggest that the progenitors of populations in northern waters, which became available for colonization only after 7000 years ago, likely dispersed from previously established populations in the Bay of Biscay.

Our genetic results also confirm dispersals of anchovies from European waters across the equator to southern Africa. The major European mtDNA lineages also occur in southern African anchovies [18], and allozyme frequencies in southern African populations are similar to those in European populations [75]. While allozyme diversities in southern African anchovies are as large, or larger, as those in European populations [6], [7], [76], both nuclear SNPs and mtDNA SNPs show lower diversity in our study. Based on a single sample of European anchovies from the NW Mediterranean, Grant & Bowen [18] suggested that similarities in allozyme and cytochrome-b frequencies with Mediterranean anchovies indicated a Mediterranean origin of present-day populations of southern African anchovies. However, our results for populations in the NE Atlantic show a closer relationship between some NE Atlantic and southern African anchovies.

### Conclusions

The analyses of multiple nDNA and mtDNA SNP markers confirm and expand previous population genetic and phylogeographic hypothesis for European anchovy populations. The nuclear SNPs yielded larger values of *F*_ST_ than did microsatellite markers and, hence, provided a greater amount of power to detect population structure. The results of our study and of several other studies demonstrate hierarchical genetic differences between populations on different temporal and spatial scales. Some divergences between populations in the Mediterranean and in the Atlantic can be explained by random drift in populations isolated by current gyres, by frontal systems, or by distance. However, some small-scale differences between populations in the Bay of Biscay and in other areas may be due to adaptive divergences in life-history traits. These life-history contrasts are ideal starting points for genomic studies to understand the genetic basis of adaptation.

Our multi-locus approach also revealed two major groups of European anchovies that are associated with different oceanic systems. One group inhabits productive wide-shelf ecosystems with physical mechanisms that enhance nutrient enrichment, an abundance of larval food and egg and larval retention, the ‘ocean triads’ [1]. Because these ecosystem features are conducive to population growth and local retention, these anchovies tend to spawn and overwinter in offshore oceanic areas, without being lost to the system. In contrast, the other group inhabits narrow-shelf areas that are dominated by upwelling. Anchovies in these populations spawn and overwinter in coastal areas to avoid offshore advection during upwelling. While the ecologies of these two groups have been described in the literature, we show here that these ecological groups are also genetically differentiated from each other to a small extent.

The analysis of the mtDNA resolves two deeply separated phylogroups in European anchovies. The frequencies of these lineages vary among populations and give insights into patterns of dispersal and colonization during the Pleistocene climate cycles. One conundrum is the genetic similarity between some anchovy populations in the Bay of Biscay and those in the NW Mediterranean. This genetic similarity can only be explained by post-glacial dispersals into these two areas by anchovies migrating from a common ancestral population. Since these are ‘wide-shelf’ populations, the ancestral refuge population may have been in the Mediterranean, or along Atlantic Africa in an area with ‘ocean triad’ characteristics. The colonization of the Bay of Biscay was followed by stepping-stone dispersals into northern coastal areas, including the English Channel and the North and Baltic seas. The genetic discontinuity at Galicia is likely due to secondary contact between the established populations in the Bay of Biscay and later secondary colonizers from southern refugia located in a narrow-shelf ocean ecosystem.

## Supporting Information

Table S1 **Summary of nuclear DNA SNP statistics in 626 samples of** ***Engraulis encrasicolus*** **by locus.** Number of alleles or haplotypes for each locus (*N*_o_ alleles), Allelic Richness (*A*) for a minimum sample size of 15 individuals, mean heterozygosity (*h*_e_) and *F*_IS_ values with their Standard Error (SE).
(DOCX)
