# Supplementary material for: Multiple SNP Markers Reveal Fine-Scale Population and Deep Phylogeographic Structure in European Anchovy (Engraulis encrasicolus L.)
Source: PLoS One. 2012 Jul 30;7(7):e42201. doi: 10.1371/journal.pone.0042201 (PMC3408476; doi:10.1371/journal.pone.0042201)
Supplement: Table S1 — Summary of nuclear DNA SNP statistics in 626 samples of Engraulis encrasicolus by locus. Number of alleles or haplotypes for each locus (N o alleles), Allelic Richness (A) for a minimum sample size of 15 individuals, mean heterozygosity (h e) and F IS values with their Standard Error (SE). (DOCX) [file pone.0042201.s001.docx]

**Supplementary Table**

**Table S1.** Summary of nuclear DNA SNP statistics in 626 samples of *E. encrasicolus* by locus. Number of alleles or haplotypes for each locus (N_o_ alleles), Allelic Richness (A) for a minimum sample size of 15 individuals, mean heterozigosity (*h*_e_) and *F*_IS_ values with their Standard Error (SE).

| SNP/Fragment | Nall | A | *h*_e_ (±SE) | *F*_IS_ (±SE) |
| --- | --- | --- | --- | --- |
| CaM-4-152 | 2 | 2.0 | 0.473 ± 0.043 | 0.083 ± 0.187 |
| CatL-7-40 | 2 | 2.0 | 0.342 ± 0.103 | -0.083 ± 0.211 |
| CK6-1-(129/276)^1^ | 2 | 2.0 | 0.186 ± 0.157 | 0.343 ± 0.301 |
| CK6-2-214 | 2 | 2.0 | 0.199 ± 0.088 | -0.014 ± 0.182 |
| KLN-235-249 | 2 | 1.8 | 0.092 ± 0.075 | -0.053 ± 0.043 |
| KLN-276-201 | 2 | 1.9 | 0.163 ± 0.082 | 0.091 ± 0.196 |
| KLN-286-(449/531)^1^ | 4 | 3.0 | 0.450 ± 0.141 | -0.033 ± 0.176 |
| KLN-287-297 | 2 | 2.0 | 0.348 ± 0.119 | 0.024 ± 0.242 |
| KLN-289-494 | 2 | 2.0 | 0.353 ± 0.125 | 0.024 ± 0.246 |
| KLN-292-351 | 2 | 2.0 | 0.203 ± 0.095 | 0.015 ± 0.216 |
| KLN-320-389 | 2 | 1.9 | 0.109 ± 0.098 | 0.179 ± 0.357 |
| KLN-323-295 | 2 | 1.9 | 0.165 ± 0.079 | 0.017 ± 0.156 |
| KLN-332-(144/444)^1, 2^ | 4 | 4.0 | 0.655 ± 0.053 | -0.045 ± 0.098 |
| KLN-337-205 | 2 | 2.0 | 0.241 ± 0.160 | 0.041 ± 0.179 |
| KLN-339-367 | 2 | 2.0 | 0.179 ± 0.096 | 0.286 ± 0.337 |
| KLN-356-139 | 2 | 2.0 | 0.262 ± 0.070 | -0.027 ± 0.133 |
| KLN-357-(474/493/506)^1^ | 5 | 3.7 | 0.603 ± 0.095 | 0.177 ± 0.162 |
| KLN-360-320 | 2 | 2.0 | 0.347 ± 0.126 | 0.046 ± 0.145 |
| KLN-369-226 | 2 | 2.0 | 0.188 ± 0.211 | -0.253 ± 0.265 |
| KLN-386-194 | 2 | 1.4 | 0.031 ± 0.053 | 0.220 ± 0.313 |
| KLN-457-303 | 2 | 1.9 | 0.129 ± 0.069 | 0.062 ± 0.303 |
| KLN-483-165 | 2 | 1.9 | 0.097 ± 0.097 | -0.041 ± 0.051 |
| KLN-491-(190/207)^1^ | 3 | 2.8 | 0.447 ± 0.059 | 0.004 ± 0.139 |
| KLN-493-(225/472)^1^ | 4 | 2.9 | 0.440 ± 0.090 | 0.030 ± 0.135 |
| KLN-496-404 | 2 | 1.9 | 0.147 ± 0.094 | 0.273 ± 0.311 |
| KLN-497-(125/158)^1^ | 4 | 3.1 | 0.579 ± 0.107 | 0.018 ± 0.145 |
| KLN-499-248 | 2 | 2.0 | 0.359 ± 0.143 | 0.166 ± 0.276 |
| KLN-505-377 | 2 | 2.0 | 0.493 ± 0.025 | -0.151 ± 0.299 |
| M3EK-44-(412/447)^1^ | 4 | 3.7 | 0.605 ± 0.168 | 0.115 ± 0.123 |
| M4-114-190 | 2 | 2.0 | 0.236 ± 0.106 | 0.026 ± 0.197 |
| M4-34-307 | 2 | 1.6 | 0.045 ± 0.054 | -0.039 ± 0.016 |
| M4-45-(265/353)^1^ | 3 | 2.9 | 0.482 ± 0.139 | 0.182 ± 0.168 |
| M4-59-278 | 2 | 1.4 | 0.031 ± 0.041 | -0.013 ± 0.023 |
| M4-76-434 | 2 | 1.8 | 0.089 ± 0.060 | -0.044 ± 0.030 |
| Rhod-127 | 2 | 2.0 | 0.177 ± 0.158 | -0.038 ± 0.088 |
| KLN-384-(212/353)+KLN-470-(403/452)^1^ | 13 | 6.3 | 0.649 ± 0.172 | 0.038 ± 0.128 |
